# Cell *In Vitro* Testing with Soil Invertebrates—Challenges and Opportunities toward Modeling the Effect of Nanomaterials: A Surface-Modified CuO Case Study

**DOI:** 10.3390/nano9081087

**Published:** 2019-07-29

**Authors:** Maria J. Ribeiro, Mónica J.B. Amorim, Janeck J. Scott-Fordsmand

**Affiliations:** 1 Department of Biology & CESAM, University of Aveiro, 3810-193 Aveiro, Portugal; 2 Department of Bioscience, Aarhus University, Vejlsovej 25, P.O. BOX 314, DK-8600 Silkeborg, Denmark

**Keywords:** earthworms, flow cytometry, coelomocytes, surface modification, safe by design, copper oxide nanoparticles

## Abstract

Soil invertebrates have been widely used in ecotoxicology studies for decades, although their use as in vitro models, albeit promising, has not been pursued as much. The immune cells of earthworms (coelomocytes) and the coelomic fluid can be used, and are a highly relevant in vitro system. Although it has been tested before, to cover the testing of nanomaterials (NMs), several challenges should be considered. NMs characteristics (dispersibility, agglomeration, etc.) can interfere with the common in vitro methodologies, not only during exposure, but also during the measurements. Here, we have assessed the effect of a CuO NMs case study using surface-modified particles, functionalized for safe-by-design strategies with ascorbate, citrate, polyethylenimine, and polyvinylpyrrolidinone, plus the pristine CuO NMs and copper chloride (CuCl_2_) for comparison. *Eisenia fetida*’s coelomocytes were exposed for 24 h via the coelomic fluid. Changes in cell viability were evaluated using flow cytometry. All materials affected the cells in a dose-related manner, where CuCl_2_ was the most toxic followed by the citrate-coated CuO NM. There was a strong correlation between NM characteristics, e.g., the hydrodynamic size, and the EC_50_ (50% Effect Concentrations) values. This screening further confirms the potential for the usage of the standard earthworm model as an in vitro standard. Further detailed in vitro studies are needed using other NMs aiming toward their implementation and standardization. Additional cell endpoints can also be assessed, making it a high content tool for mechanistic understanding.

## 1. Introduction

The current risk assessment (RA) framework for nanomaterials (NMs) still follows most of the standards previously established for conventional chemicals [1,2]. It has been long argued that these require adaptations that can reflect worst case scenarios for NMs [3,4]. The dual nature of NMs, being a particle with physical properties and also being a chemical, makes it difficult to relate the observed toxicity and its cause, and hence, the associated risks. For instance, it is not always clear how many of the ions released from metal-based NMs are the source of toxicity and how much the NMs contribute and have a specific role themselves [5,6]. Often, researchers attempt to estimate the release by measuring the ion concentration in order to differentiate between chemical (ions) and particulate toxicity, e.g., using filtration or ultra-centrifugation. However, the actual release and even the part that causes toxicity is often very difficult to measure [6]. To get a better handle on some of the toxicity issues, a favorable approach would be to have a diverse biological set of methods, each highlighting certain topics. One approach is in vitro testing, which is often faster and more cost-effective than in vivo testing [7]; in vitro testing further allows for the simultaneous screening of different parameters by focusing on the individual cell pathways of toxicity [8,9]. The majority of in vitro studies consider cells from bacteria, fish, human, or mouse-derived cellular models, and do not cover several other key organism groups. For instance, few studies deal with key terrestrial invertebrates, although they are excellent candidates for in vitro testing [9,10], especially larger sized animals. Many biological processes are conserved across mammals and invertebrates, e.g., the primary immune system, which also supports the use of invertebrates as surrogates for cross-species extrapolation to humans [11,12]. Further, the fact that these are invertebrates, hence a 3R (replacement, reduction, and refinement) compliant alternative model for laboratory testing makes them an even more important option.

Although few studies have been done, earthworms have been shown to be useful in in vitro models. For example, via exposure to Ag NMs, Hayashi et al. [13] illustrated that during in vitro exposure the biological response of *Eisenia fetida*’s coelomocytes was similar to that of human acute monocytic leukemia cell line cells (THP-1) in RPMI-1640 medium. They observed that the cytotoxicity (WST-8 assay), ROS occurrence (flow cytometry) and gene expression (quantitative PCR) responses were conserved mechanisms [13]. Bigorgne et al. [14] studied the impact of TiO_2_ particles on the coelomic cells of *E. fetida*. Other examples include the worm species, *Lumbricus terrestris* [15], where metal-specific toxicity was observed for Hg, Cd, Zn, and Pb using in vitro exposure, i.e., a high decrease in viability and phagocytic activity (Hg), lower decrease in viability, high decrease in phagocytic activity (Zn, Cd), and no decrease in viability or phagocytic activity (Pb).

However, in vitro studies also have issues, e.g., the toxicology can be far from ecological realism, especially when a variety of cell culture media (e.g., Phosphate Buffer Solution) are used that do not reflect in vivo conditions. This can be an even more important issue for NM hazard assessment, given the high reactivity and interaction with the biomolecules present in the biological fluids. The use of native fluids for cell culture (coelomic fluid) is a good approach for mimicking the real biological environment, but it is often difficult to obtain. However, the biomolecule composition will differ, and so will the interactions with the NMs and the outcome [16].

Surface modification has been widely used as a strategy to minimize NMs-biomolecule interactions in safe-by-design strategies for NM stabilization [17], but such changes will additionally influence the fate and effect of NMs [18,19,20]. For instance, coatings that enable NMs with a positive surface charge are likely to improve biocompatibility with the negatively charged cellular membrane, thus promoting cellular uptake with implications for cytotoxicity [21,22]. However, predictive risk assessment is still hampered by contradictory results that show coating-independent toxicity [23,24]. Hence, a shift in the current paradigm is necessary to cover the interactions of the NMs with the native biological fluid components, allowing for a correct prediction regarding in vivo effects.

Hence, in the present study, we have assessed the cell viability of the standard earthworm test species *Eisenia fetida* [25] using the coelomocytes and the respective coelomic fluid. Copper oxide NMs were tested, including pristine and surface-modified NMs (ascorbate, citrate, polyethylenimine, and poly(vinylpyrrolidinone), as developed in Ortelli et al. [26] as a safe-by-design strategy, plus a Cu salt (CuCl_2_) for comparison.

### 2.1. Materials and Methods

#### 2.1.1. Test Materials, Spiking, and Characterization

Pristine copper oxide nanomaterials (PRI CuO NMs) (>99% purity, PlasmaChem GmbH, Berlin, Germany, CAS No. 1317-38-0), and CuO NMs with four different surface modifications—citrate (CIT), ascorbate (ASC), polyvinylpyrrolidone (PVP), and polyethylenimine (PEI)—were used, as well as copper (II) chloride dihydrate (CuCl_2_ 2H_2_O, >99.9% purity, Sigma-Aldrich, St. Louis, MO, USA, CAS No. 10125-13-0) for comparison. Coated CuO NMs were synthetized from commercial CuO nanopowder (PlasmaChem GmbH, Berlin, Germany) and prepared according to Ortelli et al. [26]. Morphological characterization of pristine CuO NMs using Scanning Transmission Electronic Microscopy (STEM) analysis showed that CuO NMs were spherical and mono-dispersed with a primary nanoparticle average diameter of 12 ± 8 nm (N = 50) (for full characterization details, see Table S1). Stock working solutions of 10 mg Cu/L in phosphate buffered-saline (PBS: 0.01 M phosphate buffer, 0.0027 M potassium chloride, and 0.137 M sodium chloride, pH 7.4; Sigma-Aldrich, St. Louis, MO, USA, Cat. No. P4417) were used. Characterization in different media (Table 1) is provided.

The CuO NM solutions were serially diluted from stock solutions in freshly extracted coelomic fluid in the following concentrations: 0, 5, 10, 50, 100, and 500 (µg coated CuO NM)/mL; 0, 1, 5, 10, 50, 100, and 500 (µg CuO NM)/mL; and 0, 1, 5, 10, 50, and 100 µg Cu/mL for CuCl_2_. The amount of coelomic fluid was kept constant. Five replicates per treatment were used.

#### 2.1.2. Cell and Coelomic Fluid Extraction

*Eisenia fetida* (Oligochaeta, Lumbricidae) earthworms were kept in culture in OECD (Organization for Economic Cooperation and Development) artificial soil, fed ad libitum with horse manure under controlled conditions at 18 °C and a photoperiod of 16 h:8 h (light:dark). Selected organisms had similar size (300–600 mg) and developed clitellum, as described in OECD standard 222 [25]. Earthworms were carefully sampled from culture, cleaned with 1× PBS, and were transferred to a Petri dish with filter paper moistened with PBS for about 1 h for a gut purge. The posterior body part of the worms was massaged to allow expulsion of the content of the gut intestinal tract. Pools of 3–4 worms were subsequently used to obtain the cellular density, which was necessary to have enough cells for the experiment. Worms were gently placed on a glass Petri dish with sterile PBS (1 mL/worm) and an electric current was applied using a 9 V battery for six cycles of 2 s. The cell suspension was transferred to a centrifuge tube and 1% penicillin-streptomycin and 1% amphotericin was added. Cells were counted in a hemocytometer in order to obtain a density of 10^6^ cells/mL, which was seeded in siliconized tubes and left for 24 h (dark, 20 °C) to allow acclimation.

Coelomic fluid extraction, used for toxicity exposure, followed the same extraction procedure as for the cells, after which it was filtered through a 0.2 µm filter to remove cells and was supplemented with 1% penicillin-streptomycin (Sigma-Aldrich, St. Louis, MO, USA, Cat. No. P4333) and 1% amphotericin (Sigma-Aldrich, St. Louis, MO, USA, CAS# 1397-89-3) (as described by Hayashi et al. [9]). The protein concentration was measured (Biowave DNA Life Science Spectrophotometer (Biochrom Ltd., Cambridge, UK)) and set to 100 (µg protein)/mL to normalize the protein content. A control with only coelomic fluid was included, as well as a control without cells, for each treatment to verify the NMs interference. Interaction of NMs with coelomic biomolecules was allowed for 24 h (dark, 4 °C).

#### 2.1.3. In Vitro Test Procedures and Flow Cytometry

After removal of the medium using centrifugation (5 min at 1500 rpm), coelomocytes were exposed to 200 µL of each treatment for 24 h and flow cytometry analysis was carried out afterwards. Three independent assays were performed for each test material using a different pool of worms and the respective batches of coelomic fluid.

For the flow cytometry analysis, 7-aminoactinomycin D (7-AAD; Sigma-Aldrich, St. Louis, MO, USA, CAS No. 7240-37-1) and propidium iodide (PI; Sigma-Aldrich, St. Louis, MO, USA, CAS No. 25535-16-4), both DNA intercalating fluorescent dyes, were used to assess cell viability and membrane integrity. As these cannot enter cells with intact membranes, measurements will correspond to staining dead cells or cells with compromised membranes. Briefly, cells were loaded with 4 µL PI and 8 µL 7-AAD, and were immediately analyzed using flow cytometry (NovoCyte Flow Cytometer). A 488 nm laser was used for excitation; 7-AAD was detected in BL4 (675/30) and PI in BL3 (615/24). For auto-compensation, unstained and singly stained cells were processed. In each replicate, a minimum of 10,000 events were gated. To exclude the interference of NMs and debris, the solutions with each concentration and treatment were gated out of the analysis, i.e., for each cell exposure concentration, there was an equivalent exposure concentration without cells that was used for gating. Doublets were excluded using FSC-H versus FSC-A analysis [27]. Amoebocytes and eleocyte populations were identified as described in Engelmann et al. [28].

#### 2.1.4. Data Treatment

Flow cytometry data were analyzed using FlowLogic^®^ 700.2A Software (Inivai^TM^. Technologies, Mentone Victoria, Australia), and viability was normalized to the control values. Effect concentrations (ECx) were estimated by modelling data with threshold sigmoid two parameters regression models, using the Toxicity Relationship Analysis Program (TRAP v1.22, U.S. Environmental Protection Agency, Washington, DC) software.

## 3. Results

**Figure 1:**
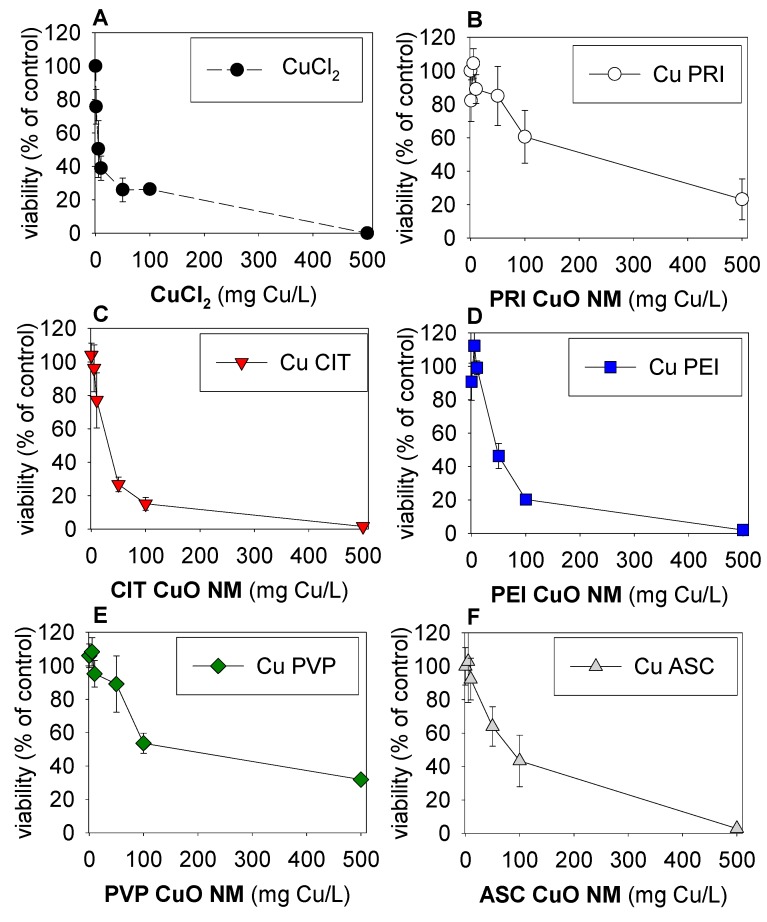
*Eisenia fetida*’s coelomocytes after 24-h exposure in coelomic fluid to 0–500 µg Cu/mL range of (**A**) CuCl_2_, (**B**) pristine (PRI) CuO NMs, and CuO NMs with different coatings: (**C**) citrate (CIT), (**D**) polyethylenimine (PEI), (**E**) polyvinylpyrrolidone (PVP), and (**F**) ascorbate (ASC). Values are expressed as % normalized to the control average ± standard error (AV±SE) (*n* = 3).

There was little or no interference with the material (without cells) and the material–cell systems when using the FSC-A-SSC-A plots, as outlined by Engelmann et al. [28]. The eleocytes were likely generally unstable (as also reported by Engelmann et al. [28]), leaving chloragosomes/debri in the lower FSC/SSC values, and this was gated out. The overlap from the particles’ spectra was also gated out. Results of the flow cytometry analysis for cell populations are shown in Figure 1. The EC_50_ (50% Effect Concentration) values (with 95% confidence intervals) were CuCl_2_: 20 (7–55) mg Cu/L, CuO NM-pristine: 197 (99–402) mg Cu/L, Cu-ascorbate: 98 (54–176) mg Cu/L, Cu-citrate: 28 (18–45) mg Cu/L, Cu-PEI: 39 (30–49) mg Cu/L, and Cu-PVP: 151 (81–284) mg Cu/L.

All materials affected the cells in a dose-related manner (Figure 1).

**Figure 2:**
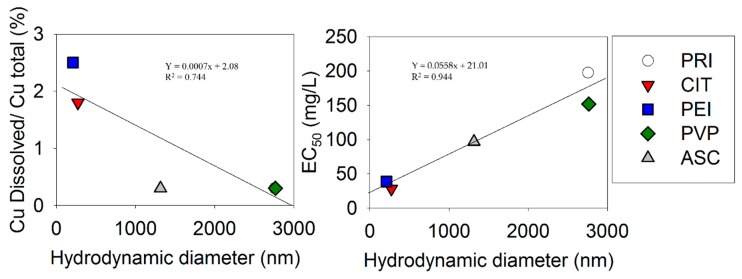
(**Left**) The Cu dissolved/CuO total ratio versus the hydrodynamic diameter in PBS. [A relationship is also observed when using surface area/volume]. (**Right**) The EC_50_ (effect concentration that causes a 50% reduction) estimate for each material versus the hydrodynamic diameter in PBS.

Figure 2 shows the relationship between the nanoparticle characteristics and the EC_50_ across materials. Only relationships with a low *p*-value are shown.

## 4. Discussion

The present study showed a clear relationship between the EC_50_ for the *Eisenia fetida*’s coelomocytes population and the particles’ characteristics, especially the hydrodynamic diameter (in PBS), which showed a high correlation (*R*^2^ = 0.94, *p* = 0.005) with the EC_50_. We also observed a correlation with NM dissolution (in Milli-Q and PBS), i.e., materials that were less dissolved were less toxic. This correlation was less strong (*R*^2^ = 0.74, *p* = 0.06) and was dominated by a more-grouped set of dissolution values, i.e., Cu dissolved/CuO total values were 0.3, 1.8, 0.3, 2.5, and 0.3. Although, the relationship with dissolution could suggest a Cu-ion related effect, it has to be considered that there was also a correlation between the hydrodynamic diameter and the Cu dissolved/CuO total (the larger the hydrodynamic diameter the less dissolved Cu was present). Hence, an alternative explanation could be, as oultined by Líbalová et al. [29], that there was a trojan-horse effect, i.e., uptake of particles and dissolution within the cells led to disintegration of the membranes. In this case, the NM accumulation may be size-related, i.e., pristine and PVP-coated particles were simply too big for an efficient phagocytosis. Obviously, our correlation could be somewhat of an artifact since our EC_50_ (measured in a coelomic fluid solution) was correlated with the hydrodynamic diameter measured in PBS. However, a similar although less strong correlation was also observed for the hydrodynamic diameter measured in Milli-Q (*R*^2^ = 0.70), but not for the hydrodynamic diameter measured in DMEM (*R*^2^ = 0.2). On the other hand, the dissolution was higher in biological media (65–69%) compared to, e.g., PBS (2.5–0.3%) and Milli-Q (0–2.8%)[26]. Hence, based on this, we would expect toxicity to be more likely to occur due to Cu-ions when using the biological fluids of earthworms. Kwak et al. [30] also observed a higher NM-dissolution in earthworm coelomic fluid than in deionized water, although this study was conducted with citrate-coated AgNMs. Therefore, this makes it likely that dissolution was also important in the present experiment. However, as mentioned, we found no correlation between Cu dissolved/CuO total in biological fluid (DMEM) and the EC_50_.

Cytotoxicity of the same ASC, CIT, PEI, and PVP-coated CuO NMs was reported in RAW264.7 macrophages with concentrations up to 60 µg Cu/mL [29]. For this study, no correlation could be found between the measured intracellular Cu and the cytotoxic effect, hence a simple interpretation of toxicity based on Cu-dissolution was rejected; rather, they suggested the trojan-horse effect. In this study, they found CuNM-PEI to be the most toxic material, which correlated somewhat with our finding (second-most toxic); however, they did not observe a correlation with the hydrodynamic diameter as we did. Differences in the results among cells types when testing various coatings were also found for Ag NMs. For instance, CIT was found to be more toxic than polyethylene glycol (PEG) [31] and PVP [32,33] in certain cell lines, but in other cell lines, there was a higher sensitivity towards PEG compared to CIT-coated NMs [34].

### In Vitro Challenges and Future Research

Flow cytometry presents data analysis challenges [3], especially when there are event count overlaps between test material, dyes, and cell signal. This means that cell signals may have to be discarded because it is not possible to discriminate between cells and particles. Flow cytometry is still one of the best techniques to provide a (more) reliable and sensitive analysis [35], but alternative dyes should be pursued to improve results when dealing with nanomaterials. Further, as pointed out by Engelmann et al. [28], cell-sorting techniques should also be included.

The coelomic fluid is a promising in vitro test media. However, this fluid is obviously less singularly repeatable in its exact content as it depends on the biology of the organisms. Nevertheless, what may be lost in precision due to this less uniform exact content of coelomic fluid over experiments may very easily be gained in accuracy (i.e., biological relevance) and hence repeatability. We have previously (Hayashi et al. [9]), as has Kwak et al. [30], showed the relevance of using physiological relevant fluids in in vitro testing, as this increased Ag NMs interaction and consequent accumulation in coelomocytes with *E. fetida*’s coelomic proteins (primarily lysenin) compared to non-native proteins. A characterization of the particles in the ceolomic fluid is obviously a prudent way forward. Finally, there are different ways to extract the fluid, e.g., using a needle, or as we have done, using a mild current. When extracting cells, there may also be some “mucus” from the body’s surface, although they were cleaned, and it is not known to what extent this happens and whether it differs across experiments. This is likely to be a similar confounding factor to that of the coelomic fluid.

## 5. Conclusions

In the present study, *Eisenia fetida*’s coemolocytes were affected by Cu-based nanomaterials. We observed a strong correlation between NMs characteristics and the EC_50_ values, especially the hydrodynamic diameter. Nevertheless, flow cytometry with NMs presents data analysis challenges, especially when there are event count overlaps between test material, dyes, and cell signal. However, flow cytometry is still one of the best techniques to provide a (more) reliable and sensitive analysis. 

## Figures and Tables

**Table 1:** Characterization of pristine and surface-modified CuO NMs samples dispersed in Milli-Q water (pH = 6.5), phosphate buffered saline (PBS) (pH = 7.4), and biological media DMEM (Dulbecco’s Modified Eagle Medium) (pH = 8.2), including ζ-potentials (mV), hydrodynamic diameter (nm), sedimentation, velocity (µm/s), and Cu_dissolved_/CuO_total_ weight ratio (%) after 24 h at 25 °C (from Ortelli et al. [26]). CIT: Citrate; ASC: Ascorbate; PVP: Polyvinylpyrrolidone; PEI: Polyethylenimine; PRI: Pristine. The reversal of the CuO pristine surface charge sign was due to the presence of the phosphate ions (PO_4_^3−^) used in the sample preparation, which were specifically adsorbed onto the CuO NMs surface.

CuO	ζ-Potential (mV)			Hydrodynamic Diameter (nm)			Sedimentation Velocity (µm/s)			Cu_dissolved_/CuO_total_ Weight Ratio (%)		
	Milli-Q	PBS	DMEM	Milli-Q	PBS	DMEM	Milli-Q	PBS	DMEM	Milli-Q	PBS	DMEM
PRI-PO_4_^3−^	−9.1 ± 0.4	−2.3 ± 2.1	−8.2 ± 7.4	1093 ± 50	2756 ± 347	55 ± 6	0.12	0.43	0.04	0.2 (1.1)	<0.3 (0.1)	67 (0.5)
CIT	−18.0 ± 0.3	−3.4 ± 1.2	−9.7 ± 0.6	368 ± 10	271 ± 43	37 ± 2	0.1	0.08	0.03	2 (0.5)	1.8 (0.4)	69 (1.0)
ASC	−17.4 ± 0.3	−8.1 ± 0.1	−9.2 ± 0.2	122 ± 1.4	1314 ± 525	73 ± 21	0.0	0.0	0.01	2 (0.5)	<0.3 (0.1)	65 (0.4)
PEI	+28.3 ± 0.7	+13.8 ± 0.1	−10.1 ± 0.7	247 ± 14	209 ± 16	45 ± 14	0.05	0.03	0.1	2.8 (0.6)	2.5 (0.6)	67 (0.5)
PVP	−8.1 ± 2.3	−0.9 ± 0.7	−9.4 ± 0.8	797 ± 84	2765 ± 432	53 ± 25	0.06	0.2	0.03	0.2 (1.0)	<0.3 (0.1)	66 (1.3)

## Supplementary Materials

The following are available online at https://www.mdpi.com/2079-4991/9/8/1087/s1, Table S1: Physical-chemical characteristics of the pristine CuO NMs.
