# Supplementary material for: Cell In Vitro Testing with Soil Invertebrates—Challenges and Opportunities toward Modeling the Effect of Nanomaterials: A Surface-Modified CuO Case Study
Source: Nanomaterials (Basel). 2019 Jul 29;9(8):1087. doi: 10.3390/nano9081087 (PMC6723996; doi:10.3390/nano9081087)
Supplement: Supplementary file 1 [file nanomaterials-09-01087-s001.pdf]

Article

Cell *In Vitro* Testing with Soil Invertebrates—Challenges and Opportunities toward Modeling the Effect of Nanomaterials: A Surface-Modified CuO Case Study

Maria J. Ribeiro [1], Mónica J.B. Amorim [1,*] and Janeck J. Scott-Fordsmand [2]

[1] Department of Biology & CESAM, University of Aveiro, 3810-193 Aveiro, Portugal
[2] Department of Bioscience, Aarhus University, Vejlsovej 25, P.O. BOX 314, DK-8600 Silkeborg, Denmark
* Correspondence: mjamorim@ua.pt; Tel.: +351-234-247-093

Received: 9 July 2019; Accepted: 18 July 2019; Published: date

Table S1: Physical-chemical characteristics of the pristine CuO NMs.

**Characteristics**	**Pristine CuO NMs**
Manufacturer	Plasma chem
CAS number	1317-38-0
Primary size distribution (average)	3 – 35 (12)
Mode (1st quartile – 3rd quartile) [nm]	10 (9.2 – 14)
Shape	Semi-spherical
Average crystallite size [nm]	9.3
Crystallite phases (%)	Tenorite 100 %
Dispersibility in water: D50 [nm];<br>Average Agglomeration Number (AAN)	135.5 ± 4.6<br>346
Dispersibility in modified MEM: D50 [nm];<br>Average Agglomeration Number (AAN)	85.2 ± 2.7<br>77
Z-potential in UP water [mV]	+28.1 ± 0.6
IsoElectric Point (pH)	10.3
Photocatalysis: photon efficiency	$1.5x10^{-4}$
Specific Surface Area [m^2 g/1]	47.0 ± 1.7
Pore sizes [nm]	13.5 ± 1.6 (BJH)<br>23.0 ± 0.9 (AVG)
Surface chemistry [atomic fraction]	Cu = 0.46±0.05: O = 0.47±0.05; C = 0.07±0.01
Chemical impurities [mg kg/1]	Na: 505 ± 30; Pb: 36 ± 2; Ag: 13 ± 4
